# Iliac Calcium Score thresholds predict cardiovascular and limb-related outcomes in TASC D aortoiliac disease

**DOI:** 10.3389/fmed.2025.1655229

**Published:** 2025-09-17

**Authors:** António Pereira-Neves, João Rocha-Neves, Tiago Costa-Pereira, Hugo Ribeiro, Lara R. Dias, Luís Duarte-Gamas, José P. Andrade

**Affiliations:** ^1^Unit of Anatomy, Department of Biomedicine, Faculty of Medicine, University of Porto, Porto, Portugal; ^2^Local Health Unit São João, Department of Angiology and Vascular Surgery, Porto, Portugal; ^3^CINTESIS@RISE, Unit of Anatomy, Department of Biomedicine, Faculty of Medicine, University of Porto, Porto, Portugal; ^4^Local Health Unit Alto Ave, Department of Angiology and Vascular Surgery, Guimarães, Portugal; ^5^Department of Surgery and Physiology, Faculty of Medicine, University of Porto, Porto, Portugal; ^6^Community Palliative Care Team Gaia, Local Health Unit Gaia and Espinho, Vila Nova de Gaia, Portugal; ^7^Faculty of Medicine, University of Coimbra, Coimbra, Portugal; ^8^Coimbra Institute for Biomedical Research, Coimbra, Portugal; ^9^Department of Community Medicine, Health Information and Decision, Faculty of Medicine, University of Porto, Porto, Portugal; ^10^Local Health Unit Tâmega e Sousa, Department of Angiology and Vascular Surgery, Penafiel, Portugal

**Keywords:** peripheral artery disease, arterial calcification, TASC II, revascularization, amputation, cardiovascular outcomes

## Abstract

**Introduction:**

Extensive lower limb arterial calcification complicates revascularization and is linked to poor outcomes, including limb loss and cardiovascular events. Standardized scoring systems are lacking, particularly in aortoiliac TASC II D lesions. This study evaluated the prognostic value of a CT-based Iliac Calcium Score (ICS) in predicting major adverse limb events (MALE), cardiovascular events (MACE), and all-cause mortality in patients with severe aortoiliac disease.

**Methods:**

In this prospective cohort (2013–2024), 109 patients with TASC II D aortoiliac occlusive disease underwent elective revascularization and preoperative CT angiography. Iliac artery calcification was scored semiquantitatively by morphology, circumference, and lesion length. Patients were stratified into low (≤ 36) and high (≥ 37) ICS groups. Outcomes included MALE, MACE, and mortality, analyzed using Kaplan-Meier and Cox regression.

**Results:**

The study included 109 patients (95.4% male) with a median follow-up of 67 months. Baseline characteristics were similar across ICS groups, though ICS ≥ 37 was associated with more advanced Rutherford stages (*p* = 0.035). At 30 days, both groups improved clinically, but Rutherford class improvement was greater in the ICS ≤ 36 group (*p* = 0.013), with no other significant differences. At 1 year, MALE was more frequent in patients with ICS ≥ 37 (48.1% vs. 27.3%; *p* = 0.024). At 60 months, this group showed significantly lower amputation-free (74.5% vs. 97.8%; *p* = 0.002), MACE-free (47.3% vs. 73.4%; *p* = 0.005), and overall survival (54.6% vs. 77.0%; *p* = 0.013). Acute heart failure occurred only in the high ICS group (*p* = 0.015), while patency rates were similar. ICS ≥ 37 remained an independent predictor of MACE (aHR 2.30; *p* = 0.008) and major amputation (aHR 7.52; *p* = 0.008) in multivariable analysis.

**Conclusion:**

In patients with extensive TASC II D aortoiliac occlusive disease, an ICS ≥ 37 was independently associated with increased long-term risk of MACE, MALE, and reduced overall survival, despite similar short-term outcomes. These findings support the integration of preoperative calcium scoring as a simple, lesion-specific tool for risk stratification, procedural planning, and personalized postoperative surveillance in complex peripheral arterial disease.

## Highlights

- Calcium score ≥ 37 independently predicted limb loss and cardiovascular mortality, with a 7.5-fold higher amputation risk and 2.3-fold higher major adverse cardiovascular event risk (*p* < 0.01).
- Late divergence in survival curves (e.g., acute heart failure and mortality after 24 months) suggests calcification may contribute to progressive systemic vascular dysfunction.
- Primary/secondary patency pattern similarities suggest that calcification impacts long-term clinical outcomes more than procedural success, highlighting the need for post-intervention monitoring.

## Introduction

Coronary artery calcium (CAC) scoring has become a cornerstone in coronary heart disease risk stratification, with robust evidence supporting its incremental prognostic value beyond traditional risk factors and also for cardiovascular risk stratification (1, 2). Moreover, a systematic review and meta-analysis demonstrated that CAC scoring improves discrimination for cardiovascular events [pooled C-statistic gain: 0.036 (95% CI 0.020–0.0520)] while highlighting limitations in cost-benefit ratios for routine use (3). Coronary artery calcium (CAC) scores were significantly associated with biological aging, with scores > 400 adding up to 30 years to predicted age in younger individuals, while scores < 10 reduced biological age by up to 10 years in older patients; calcium-adjusted age reclassified 55% of low-risk and 45% of intermediate-risk individuals (by Framingham score) into higher-risk categories, improving mortality prediction beyond observed age (*p* < 0.0001) (4).

In peripheral artery disease (PAD), extensive aortoiliac occlusive disease, classically classified as TransAtlantic Inter-Society Consensus (TASC) II type D aortoiliac lesions, presents unique challenges. While open surgery remains the gold standard, endovascular and hybrid approaches show comparable mid-term patency rates (85% at 5 years) with lower perioperative risks in high-comorbidity patients (5–7). In this complex aortoiliac disease, characterized by extensive occlusions or calcifications involving the distal aorta and bilateral iliac arteries, vascular calcification exacerbates technical challenges during revascularization, especially with endovascular techniques.

The pathophysiology of lower limb arterial calcification (LLAC) in aortoiliac disease involves both intima and media layers, with distinct clinical implications. While intimal calcification is classically linked to atherosclerosis and inflammatory processes, predominating in patients with traditional risk factors (e.g., smoking, hyperlipidemia), medial calcification is systematically regulated and linked to diabetes, chronic kidney disease (CKD), and aging-related reduction in arterial compliance, exacerbating hemodynamic impairment and limb ischemia (8–11).

In the iliac arteries, calcification predominantly localizes to the intima in atherosclerotic plaques, but medial calcification coexists in patients with metabolic comorbidities, amplifying cardiovascular risk (9, 10). This dual calcification pattern mirrors findings in femoral and tibial arteries, where medial calcification correlates strongly with advanced ischemia severity and limb-related morbidity (11–13).

Emerging evidence suggests that scoring LLAC could refine PAD management. Studies in PAD populations associate elevated calcium scores with limb-related events, including major amputations (12, 14), but also with cardiovascular events, including all-cause mortality (15).

Despite its clinical relevance to revascularization outcomes and amputation risk, LLAC lacks standardized quantification. Current methods range from qualitative visual scores like Peripheral Arterial Calcium Scoring System (PACSS) to adapted Agatston protocols, creating heterogeneity in research and clinical applications (9, 14, 16, 17). Furthermore, despite their complexity and clinical relevance, no validated calcium scoring system currently exists for TASC II type D aortoiliac lesions.

This study aims to evaluate the prognostic value of a standardized lower limb calcium scoring system in predicting outcomes such as major adverse limb events (MALE), MACE, and all-cause mortality, helping to optimize risk stratification and guide therapeutic decisions in this high-risk population.

## Materials and methods

### Patient selection

A total of 180 consecutive patients who underwent elective aortoiliac revascularization between January 2013 and January 2024 were included in a prospective cohort. All patients were selected from a tertiary and a community hospital and had aortoiliac TASC II type D lesions, excluding those with aortoiliac aneurysmatic disease (18). The choice between open surgery and an endovascular procedure was made by a shared decision between the patient and the surgeon, considering the surgeon and the institution’s experience and preferences.

**FIGURE 1:**
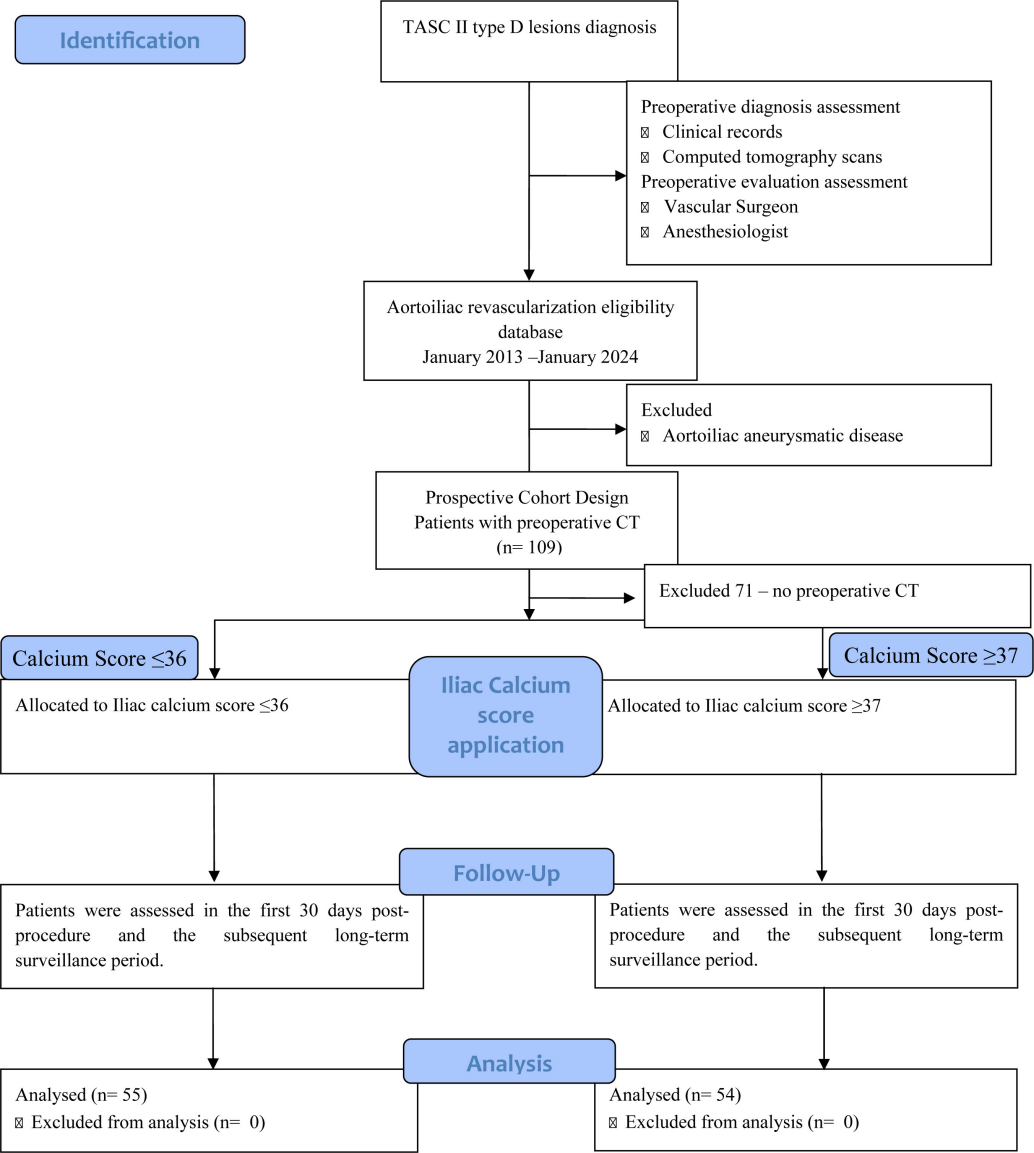
Flowchart of patient selection and cohort allocation.

A *post hoc* analysis was performed, including only patients who had undergone preoperative computed tomography (CT). Of the initial 180 patients, only 109 had preoperative CT and were included in the final analysis (Figure 1). Among baseline comorbidities, the only statistically significant difference between included and excluded patients was a higher prevalence of smoking in the included cohort (95.4% vs. 85.9%, *p* = 0.024) (Supplementary Table 1).

The patients’ demographic and clinical profiles — encompassing cardiovascular risk factors as well as procedural and lesion-specific information — were obtained through a review of their medical records, including data on whether a CT scan had been performed (7). Further information regarding the type of lesion is described elsewhere (18). Patients underwent evaluation during the initial 30 days following the procedure and throughout the subsequent long-term follow-up period, which included regular clinical and hemodynamic assessments in an outpatient setting. This study is under the framework of the Strengthening the Reporting of Cohort Studies in Surgery (STROCSS) 2019 Guideline (19).

The local Ethics Committee approved the study protocol and respects the Declaration of Helsinki. Patient informed consent was handled accordingly, and all data processing was anonymous (Protocol Number 246-18). It respects the European Union General Data Protection Regulation (GDPR).

### Definitions and outcomes

Retrieved data was registered in agreement with the *Reporting Standards* of the *Society for Vascular Surgery* for lower extremity ischemia (20). The Rutherford chronic ischemia classification was used to classify the symptoms and severity of chronic lower extremity ischemia (21).

The primary outcome was long-term MACE, defined as a composite outcome, including myocardial infarction (MI), acute heart failure (AHF), and all-cause mortality (22). MALE was described as a composite of reintervention, including reintervention due to primary assisted patency, secondary patency, major amputation of the revascularized artery segment, and occlusion without intervention (23).

### Imagiological parameters

Iliac artery calcification was quantified by two independent operators using a validated CT-based semiquantitative scoring system, which evaluates three distinct features: morphology, circumferential involvement, and length of calcification. Axial CT images with 5 mm slice thickness and bone window settings (width 3077 HU; level 570 HU) were analyzed by experienced vascular surgeons and non-contrast CT slices acquired before contrast injection were used for quantification. Each iliac arterial segment (common and external, bilaterally) was scored separately. Morphology was graded from 0 to 3, with 0 indicating no calcification, 1 representing thin linear calcifications ≤ 1 mm (“eggshell” type), two corresponding to thick linear calcifications > 1 mm with convex margins, and 3 indicating bulky calcifications > 2 mm with convex luminal margins. Circumferential and length involvement were each scored from 0 to 4, based on percentage involvement (0 = none; 1 = 1%–25%; 2 = 26%–50%; 3 = 51%–75%; 4 = 76%–100%). The highest value in each segment was recorded (24). The final score by patient was calculated by adding the scores from each of the four iliac segments.

### Statistical analysis

The sample size for a survival test was calculated using online Sample Size Calculators for Designing Clinical Research,^1^ with a statistical power (β) of 80% and a significance level of 0.05. The sample size (95 patients) was estimated based on an expected hazard ratio of 2, for cardiovascular events, between groups and a projected 80% event-free survival rate at the end of follow-up (7, 25, 26).

Statistical analyses were performed using SPSS (IBM Corp., released 2023. IBM SPSS Statistics for Windows, version 29.0.2.0, Armonk, NY, United States). Continuous variables were assessed for normality using histograms and skewness. Normally distributed variables were reported as mean ± standard deviation (SD), and comparisons between groups were performed using the independent samples *t*-test. Non-normally distributed continuous variables were reported as median and interquartile range (IQR), and compared using the Mann-Whitney U test. When appropriate, categorical variables were presented as frequencies and percentages and compared using the chi-square or Fisher’s exact test.

To facilitate the interpretation and clinical applicability of the Iliac Calcium Score (ICS), dichotomization was performed using visual binning rather than Receiver Operating Characteristic (ROC) analysis. A threshold of 36.5 was selected based on observed data distribution and clinical reasoning, corresponding to the point of greatest contrast in event rates (MACE) between groups. This cut-off was then used to stratify patients into low (≤ 36) and high (≥ 37) ICS groups for further analysis.

Kaplan-Meier survival analyses were conducted to evaluate time-to-event outcomes, including MACE, MALE, and all-cause mortality. Log-rank tests were used to compare survival curves between groups.

The Log Rank estimator was utilized to test the effect of the ICS on time-dependent variables. Multivariate Cox regression analysis was performed for independent predictors of long-term MACE and all-cause mortality, using the backward stepwise regression method. The backward stepwise regression method was applied, and variables with *p* < 0.20 were included.

All statistical tests were two-sided; a *p*-value < 0.05 was considered statistically significant.

## Results

### Population data

The cohort included 109 patients with a median follow-up of 67 (IQR 56.2–77.8) months. Table 1 presents the baseline characteristics of the patients included, stratified by ICS (≤ 36 vs. ≥ 37), with 55 patients and 54 patients, respectively. Most patients were male (95.4%), with similar distribution between groups (92.7% vs. 98.1%; *p* = 0.363). Although the two groups did not differ significantly in most comorbidities, patients with higher calcium scores (≥ 37) presented a trend for hypertension (77.8% vs. 61.8%; *p* = 0.070) and had a higher prevalence of chronic kidney disease (13.0% vs. 5.5%; *p* = 0.175) and chronic heart failure (CHF) (13.0% vs. 5.5%; *p* = 0.175), albeit without reaching statistical significance.

**TABLE 1:** Patient’s demographics and comorbidities.

Characteristics	Total *n* = 109 (%)	Iliac Calcium Score ≤ 36 *n* = 55	Iliac Calcium Score ≥ 37 *n* = 54	*P*-value
Age, years (mean ± SD)	62.0 ± 8.70	59.8 ± 8.55	64.2 ± 8.33	0.989
Gender, male	104 (95.4)	51 (92.7)	53 (98.1)	0.363
Smoking history	104 (95.4)	54 (98.2)	50 (92.6)	0.206
Hypertension	76 (69.7)	34 (61.8)	42 (77.8)	0.070
Dyslipidemia	79 (72.5)	39 (70.9)	40 (74.1)	0.711
Diabetes	29 (26.6)	12 (21.8)	17 (31.5)	0.254
CKD	10 (9.2)	3 (5.5)	7 (13.0)	0.175
CAD	29 (26.6)	12 (21.8)	17 (31.5)	0.254
CVD	16 (14.7)	8 (14.5)	8 (14.8)	0.968
COPD	12 (11.0)	7 (12.5)	5 (9.3)	0.563
CHF	10 (9.2)	3 (5.5)	7 (13.0)	0.175
**ASA**				
III	46 (42.2)	23 (41.8)	23 (42.6)	0.356
IV	58 (53.2)	31 (56.4)	27 (50.0)	
V	5 (4.6)	1 (1.8)	4 (7.4)	
**Rutherford classification**				
3	28 (25.7)	18 (32.7)	10 (18.5)	**0.035**
4	45 (41.3)	24 (43.6)	21 (38.9)	
5	29 (26.6)	13 (23.6)	16 (29.6)	
6	7 (6.4)	0 (0.0)	7 (13.0)	
Open surgery	70 (64.2)	39 (70.9)	31 (57.4)	0.103
Endovascular technique	29 (26.6)	14 (25.5)	15 (27.8)	
Hybrid surgery	10 (9.2)	2 (3.6)	8 (14.8)	
ABI, (mean ± SD)	0.30 ± 0.120	0.31 ± 0.133	0.29 ± 0.107	0.065

Variables are presented as *N* (%) unless otherwise specified. ABI, Ankle-brachial index (preoperative); ASA, American Society of Anesthesiologists Physical Status Classification System; CAD, coronary artery disease; CHF, chronic heart failure; CKD, chronic kidney disease (creatinine = 1.5 mg/dl); COPD, chronic obstructive pulmonary disease; CVD, cerebrovascular disease; SD, standard deviation. Bold value represents statistically significant results (*p* < 0.05).

Notably, a significant difference was observed in the distribution of Rutherford classification (*p* = 0.035), with advanced stages (V and VI) being more frequent in the higher calcium score group. Overall, approximately 75% of the cohort presented with chronic limb-threatening ischemia.

The majority of procedures were open surgery (64.2%), followed by endovascular (26.6%) and hybrid (9.2%), with no significant differences between ICS groups (*p* = 0.103). The mean preoperative ABI was 0.30 ± 0.120, slightly higher in the ICS ≤ 36 group (0.31 ± 0.133) than in the ICS ≥ 37 group (0.29 ± 0.107; *p* = 0.065).

### Thirty-day outcomes according to ICS

Table 2 summarizes the 30 days outcomes. The mean change in ABI was 0.43 ± 0.235 (0.48 ± 0.241 in ICS ≤ 36 vs. 0.37 ± 0.218 in ICS ≥ 37; *p* = 0.167). Although both groups showed clinical improvement in the Rutherford chronic ischemia classification (Δ = −2.6 ± 1.41), patients with lower calcium scores exhibited a significantly greater mean improvement (Δ = −2.9 ± 1.10 vs. −2.4 ± 1.61, *p* = 0.013). Median intensive care unit (ICU) stay was 2 days (IQR 0–3), with no significant difference between groups (*p* = 0.710). Median ward stay was seven days (IQR 4–17.75), also similar between groups (*p* = 0.131).

**TABLE 2:** Patient’s 30 days outcomes according to iliac calcium score.

	Total *n* = 109 (%)	Iliac Calcium Score ≤ 36 *n* = 55	Iliac Calcium Score ≥ 37 *n* = 54	*P*-value
AKI	13 (11.9)	6 (10.9)	7 (13.0)	0.765
ABI Δ, (mean ± SD)	0.43 ± 0.235	0.48 ± 0.241	0.37 ± 0.218	0.167
Rutherford Δ, (mean ± SD)	2.6 ± 1.41	−2.9 ± 1.10	−2.4 ± 1.61	**0.013**
ICU (days), (median–IQR)	2 (0–3)	2 (0.5–3)	1 (0–3)	0.710*
Ward (days), (median–IQR)	7 (4–17.75)	6 (3–11.5)	10 (4–21)	0.131*
MINS	19 (17.4)	9 (16.4)	10 (18.5)	0.645
MALE	24 (22.0)	9 (16.4)	15 (27.8)	0.150
MACE	11 (10.1)	7 (12.7)	4 (7.4)	0.357
All cause-mortality	6 (5.5)	4 (7.3)	2 (3.7)	0.679

*Non-parametric test. Variables are presented as *N* (%) unless otherwise specified. ABI, Ankle-brachial index Δ - postoperative minus preoperative; AKI, Acute kidney injury; Rutherford chronic ischemia Δ, preoperative minus postoperative; MACE, major adverse cardiovascular event; MALE, major adverse limb event; MINS, myocardial injury after non-cardiac surgery;ICU, in stay on intensive care unit; IQR, interquartile range. Bold value represents statistically significant results (*p* < 0.05).

The incidence of acute kidney injury (AKI) was 11.9% overall (10.9% vs. 13.0%; *p* = 0.765). The incidence of myocardial injury after non-cardiac surgery (MINS) was 17.4% (16.4% vs. 18.5%; *p* = 0.645), MALE 22.0% (16.4% vs. 27.8%; *p* = 0.150), MACE 10.1% (12.7% vs. 7.4%; *p* = 0.357), and all cause-mortality 5.5% (7.3% vs. 3.7%; *p* = 0.679).

### One-year outcomes according to ICS

Table 3 shows the 1 year outcomes. Prosthetic infection occurred in 5.5% of patients, equally distributed between groups (5.5% vs. 5.5%; *p* = 0.981). Patients with higher ICS (≥ 37) experienced a significantly greater incidence of MALE compared to those with lower scores (48.1% vs. 27.3%, *p* = 0.024). Although higher rates of MACE and all-cause mortality were observed in the high ICS group (24.1% vs. 16.4% and 20.4% vs. 12.7%, respectively), these differences were not statistically significant (*p* = 0.316 and *p* = 0.283, respectively).

**TABLE 3:** Patient’s 1 year outcomes according to iliac calcium score.

	Total *n* = 109 (%)	Iliac Calcium Score ≤ 36 *n* = 55	Iliac Calcium Score ≥ 37 *n* = 54	*P*-value
Prosthetic infection	6 (5.5)	3 (5.5)	3 (5.5)	0.981
MALE	41 (37.6)	15 (27.3)	26 (48.1)	**0.024**
MACE	22 (20.2)	9 (16.4)	13 (24.1)	0.316
All cause-mortality	18 (16.5)	7 (12.7)	11 (20.4)	0.283

Variables are presented as *N* (%) unless otherwise specified. MACE, major adverse cardiovascular event; MALE, major adverse limb event. Bold value represents statistically significant results (*p* < 0.05).

### Multivariable analysis of predictors for MACE and major lower limb amputation

Table 4 presents the Cox proportional hazards regression analysis results for MACE and major lower limb amputation (MLLA).

**TABLE 4:** Cox multivariable regression proportional hazard ratio for major adverse cardiovascular events and major lower limb amputation.

	Non-adjusted hazard ratios	95% confidence interval	*P*-value	Adjusted hazard ratios	95% confidence interval	*P*-value
**MACE**						
Hypertension	1.325	0.768–2.286	0.312	NC	–	–
CKD	2.708	1.478–4.962	< 0.001	NC	–	–
CHF	3.485	1.954–6.215	< 0.001	2.604	1.147–5.908	0.022
ICS ≥ 37	2.310	1.256–4.250	0.007	2.296	1.246–4.229	0.008
ICS (cont)	1.035	1.005–1.066	0.021	1.036	1.006–1.068	0.020
**MLLA**						
Hypertension	2.424	0.714–8.242	0.156	NC	–	–
CKD	2.521	0.841–7.560	0.099	NC	–	–
CHF	1.693	0.494–5.803	0.402	NC	–	–
ICS ≥ 37	7.521	1.693–33.409	0.008	7.521	1.693–33.409	0.008
ICS (cont)	1.142	1.030–1.26	0.011	1.142	1.030–1.26	0.011

CHF chronic heart failure; CKD, chronic kidney disease (creatinine = 1.5 mg/dl); ILC, Iliac Calcium Score; MACE, major adverse cardiovascular event; MLLA, major lower limb amputation; NC, not confirmed; cont, continuous variable.

For MACE, unadjusted analysis revealed that CKD (HR 2.708, 95% CI 1.478–4.962, *p* < 0.001), CHF (HR 3.485, 95% CI 1.954–6.215, *p* < 0.001), and an ICS ≥ 37 (HR 2.310, 95% CI 1.256–4.250, *p* = 0.007) were significant predictors. After adjustment, CHF (adjusted HR 2.604, 95% CI 1.147–5.908, *p* = 0.022) and calcium score ≥ 37 (adjusted HR 2.296, 95% CI 1.246–4.229, *p* = 0.008) remained independently associated with increased MACE risk.

For MALE, CHF was a significant predictor (adjusted HR 2.116, 95% CI 1.105–4.052, *p* = 0.024), while calcium score did not reach statistical significance in the adjusted model.

For MLLA, ICS ≥ 37 was strongly associated with increased risk both in unadjusted (HR 7.521, 95% CI 1.693–33.409; *p* = 0.008) and adjusted models (aHR 7.521, 95% CI 1.693–33.409; *p* = 0.008).

The continuous calcium score variable was also significant for MACE (aHR 1.035, 95% CI 1.005–1.066; *p* = 0.021) and MLLA (aHR 1.142, 95% CI 1.030–1.260; *p* = 0.011).

### Survival analysis

#### Limb-related outcomes

**FIGURE 2:**
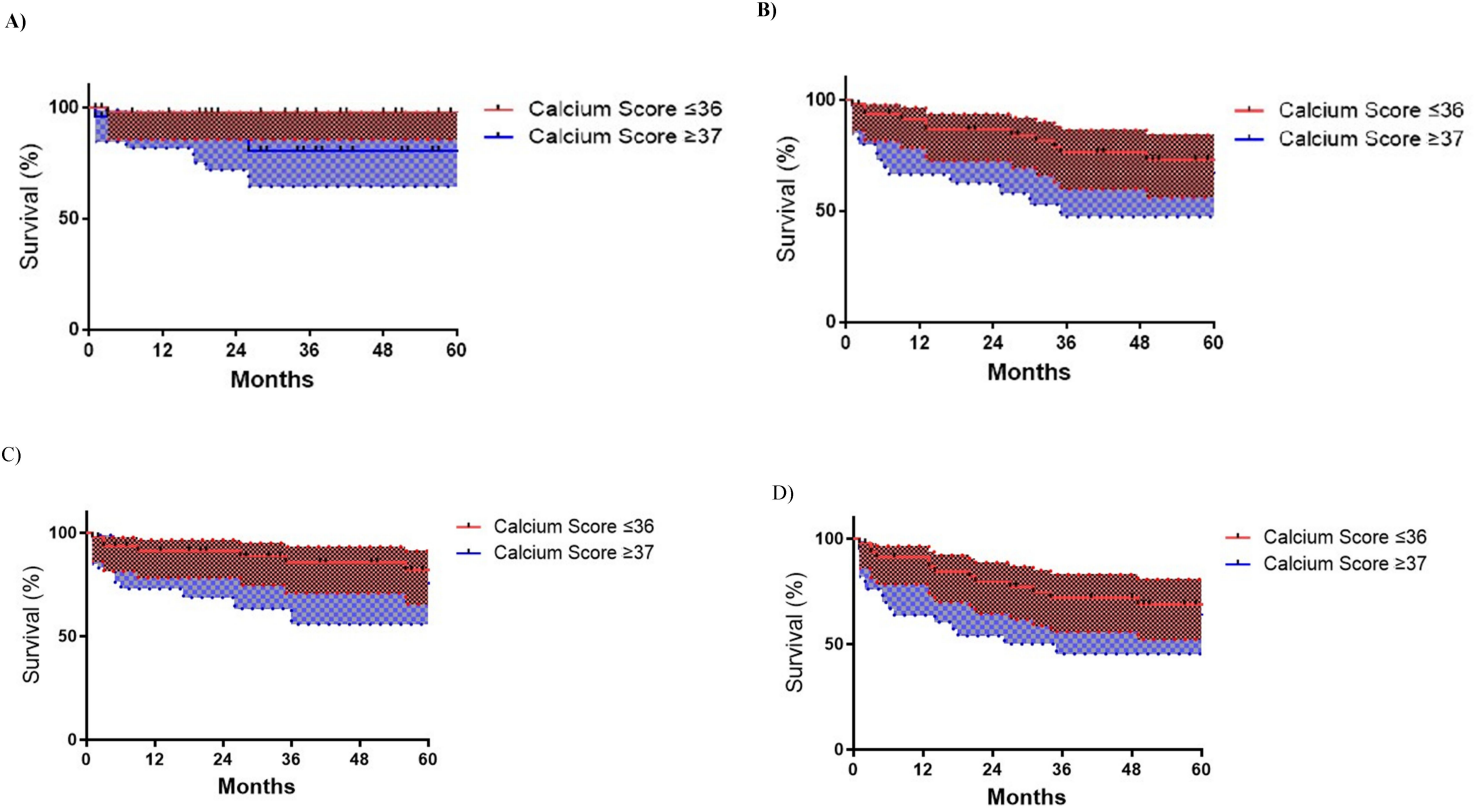
Kaplan-Meyer limb outcomes survival curves according to Iliac Calcium Score cut-off ≥ 37 status. **(A)** Major Lower limb amputation (MLLA) (*p* = 0.002); **(B)** Primary patency (*p* = 0.193); **(C)** Secondary patency (*p* = 0.233); **(D)** Major adverse limb events (MALE) (*p* = 0.139).

Kaplan-Meier analysis revealed significantly lower amputation-free survival in patients with calcium scores ≥ 37 vs. ≤ 36 (log-rank *p* = 0.002), with 60 months rates of 74.5% vs. 97.8% (Figure 2A). MALE trended higher in the ≥ 37 group (50.8% vs. 62.5% survival; *p* = 0.139) (Figure 2D), while primary/secondary patency rates showed no significant differences (Figures 2B, C).

#### Cardiovascular outcomes

**FIGURE 3:**
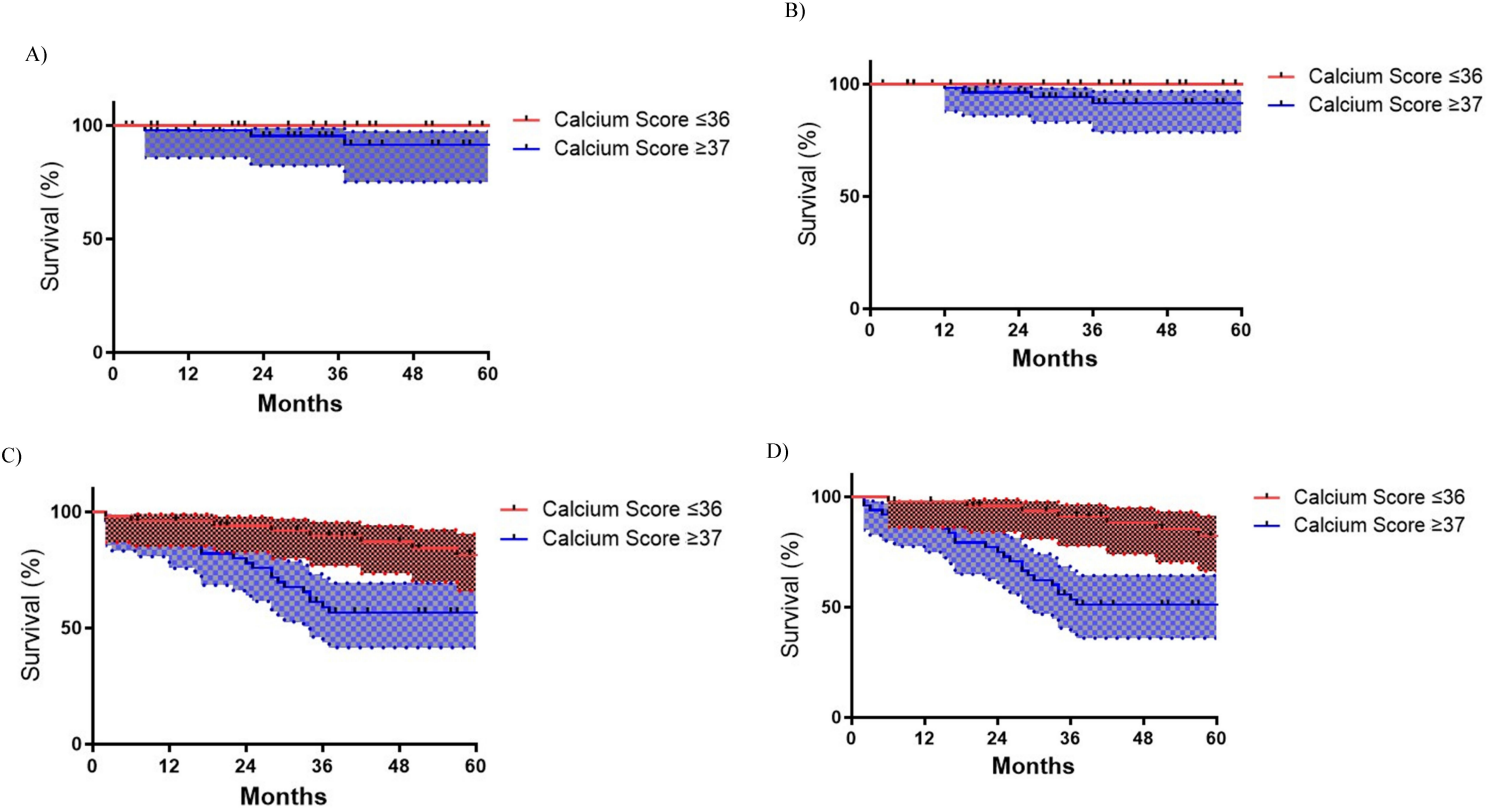
Kaplan-Meyer cardiovascular outcomes survival curves according to Iliac Calcium Score cut-off ≥ 37 status. **(A)** Acute myocardial infarction (AMI) (*p* = 0.407); **(B)** Acute heart failure (AHF) (*p* = 0.015); **(C)** All-cause mortality (*p* = 0.013); **(D)** Major adverse cardiac events (MACE) (*p* = 0.005).

Patients with ICS ≥ 37 exhibited significantly worse cardiovascular outcomes, including a higher risk of MACE (log-rank *p* = 0.005), with 60-month MACE-free survival rates of 47.3% compared to 73.4% in the ≤ 36 group (Figure 3D) and higher risk of all-cause mortality (log-rank, *p* = 0.013), with 60-month survival rates of 54.6% versus 77.0% (Figure 3C). Notably, AHF occurred exclusively in the ≥ 37 group, where AHF-free survival at 60 months was 87.7% compared to 100% in the ≤ 36 group (*p* = 0.015) (Figure 3B). Acute myocardial injury (AMI) rates showed no significant differences (Figure 3A).

## Discussion

This study demonstrates that a standardized ICS ≥ 37 could be considered a prognostic marker for 1 year and midterm adverse limb and cardiovascular outcomes in patients with TASC D aortoiliac disease undergoing revascularization. However, calcium burden does not seem to significantly impact short-term survival and perioperative complications. Elevated calcium burden was independently associated with a 2.3-fold increased risk of MACE and a striking 7.5-fold higher risk of major lower limb amputation over time. The threshold effect was particularly pronounced in longitudinal outcomes, with patients scoring ≥ 37 in the ICS exhibiting significantly reduced amputation-free survival (74.5% vs. 97.8% at 60 months) and cardiovascular event-free survival (47.3% vs. 73.4%). Notably, calcium burden correlated with advanced baseline ischemia severity and attenuated clinical improvement in Rutherford classification, underscoring its dual role as both a marker of anatomical complexity and a predictor of functional recovery.

The findings that higher calcium scores are associated with more advanced Rutherford stages (5 and 6) are consistent with previous studies that have identified a link between arterial calcification and the severity of limb ischemia. In a study of 116 patients, increasing Rutherford categories were independently associated with higher calcification scores, even after adjusting for cardiovascular risk factors and the extent of occlusive disease (11). Similarly, another study demonstrated that patients with Rutherford stages 5–6 had significantly higher iliac calcification scores compared to those with milder ischemia (stages 1–2) (13). Huang et al. (8) also reported that patients with more advanced Fontaine stages tended to have significantly higher calcification scores, further supporting the association between calcification burden and clinical severity. These findings reinforce the prognostic significance of calcium scoring in assessing lower limb ischemia.

At 30 days follow-up, the cohort with higher calcification score demonstrated attenuated improvement in Rutherford classification post-revascularization, with no significant differences in other limb-related outcomes or adverse cardiovascular events. These results are partially consistent with those of Megale et al. (12), who also reported no significant association between aortic calcium scores and 30 days mortality (*p* = 0.679), patency (*p* = 0.167), or technical success (*p* = 0.103) across 14 interventions in 11 patients. However, in contrast to our findings, Megale et al. observed a significantly higher calcium score in patients who required amputation (5,767.6 vs. 805.3; *p* = 0.02) or underwent subsequent revascularization (3,686.8 vs. 645.2; *p* = 0.008) within 30 days. These comparisons suggest that while calcification burden may influence early clinical recovery, its association with short-term major events remains inconsistent.

While no procedural preference differences were found (*p* = 0.103), this may reflect center-specific strategies rather than calcium’s technical irrelevance. Our cohort’s high open surgery rate (64.2%) might mask this relationship, as bypass grafting may circumvent calcification-related challenges. While Megale et al. (15) also did not find any statistical difference, Kang et al. on the other hand, in a cohort with 124 patients, reported that technical success of infra-popliteal angioplasty was significantly lower in patients with extensive tibial artery calcification (71.1%) compared to those with minimal (95.3%) or intermediate calcification (91.7%) (*p* = 0.001), with extensive calcification emerging as an independent predictor of technical failure (*p* = 0.014) (10).

In this study, no significant differences were observed in primary or secondary patency rates between patients with high versus low calcium scores, both in short-term (30 days) and long-term follow-up (up to 60 months), which may reflect our high open surgery rate (64.2%) but aligning with the findings of Megale et al., which included patients submitted to both open and endovascular techniques, who also reported no significant association between calcium score and patency at any time point (12, 15). However, a more anatomically focused study which included 733 limbs submitted to drug-coated balloon (DCB) angioplasty for *de novo* femoropopliteal lesions, demonstrated that femoropopliteal lesions with bilateral calcification ≥ 5 cm [peripheral artery calcification scoring system (PACSS) 4] had significantly lower 1 year primary patency (82.6%; *p* < 0.001), with PACSS 4 emerging as an independent predictor of restenosis (HR 1.82; 95% CI 1.15–2.87; *p* = 0.010), suggesting that the pattern and extent of calcification may play a relevant role in long-term durability of revascularization with DCB (27).

Regarding amputation, this study data showed robust association with a significantly lower major amputation-free survival in patients with calcium scores ≥ 37 (74.5% vs. 97.8% at 60 months; log-rank *p* = 0.002), in agreement with previous studies linking extensive calcification to worse limb outcomes, albeit some exceeding rates likely reflecting our cohort’s critical ischemia severity. For instance, Huang et al. (8) reported a 2.88-fold increased risk of amputation in patients in the highest calcium score quartile (95% CI 1.18–12.72; *p* = 0.03), and Kang et al. (10) found that patients with extensive tibial calcification had significantly lower 2 years amputation-free survival (Extensive Calcification: 58.9% vs. Minimal Calcification: 79.0% vs. Intermediate Calcification: 95.3%; *p* < 0.001), with extensive calcification being an independent predictor (HR 9.90; 95% CI 2.05–47.75; *p* = 0.004). Similarly, Dong et al. (28) identified tibial artery annular calcification as an independent predictor of unplanned amputation (HR 3.74; 95% CI 1.71–8.19; *p* = 0.001), reinforcing the importance of the calcification pattern.

In terms of MALE, a significantly higher incidence was observed at 1 year in patients with higher calcium scores (48.1% vs. 27.3%; *p* = 0.024), although this association did not remain significant in adjusted analysis. These results likely reflect the persistent significance of major amputations over time, while the lack of sustained differences in primary/secondary patency rates may have diluted the composite MALE outcome’s statistical significance in adjusted models. This suggests that calcification’s strongest prognostic impact, measured through ICS, lies in irreversible limb loss rather than revascularization-dependent endpoints in the aortoiliac population, and since distal vessel disease were not systematically included in the analysis, ICS may serve as a reflection of the overall systemic arterial calcification burden, including in the lower limbs. This trend is supported by findings from Megale et al., where higher below-the-knee calcium scores were associated with MALE both at 30 days (679.4 vs. 158.9; *p* = 0.019) and 1 year (910.1 vs. 12.3; *p* = 0.002) (12).

Patients with higher ICS (≥ 37) experienced significantly worse cardiovascular outcomes over time in this cohort. At 5 years, MACE-free survival was markedly lower in the high calcium group compared to those with lower scores (47.3% vs. 73.4%; log-rank *p* = 0.005), and all-cause mortality was also higher (54.6% vs. 77.0%; log-rank *p* = 0.013). Notably, acute heart failure (AHF) occurred exclusively in the high calcium group, with 60 months heart failure-free survival rates of 87.7% versus 100% in the lower calcium group (*p* = 0.015). Although rates of acute myocardial injury did not differ significantly between groups, as opposite to AHF and all-cause mortality, multivariable analysis confirmed that a calcium score ≥ 37 remained an independent predictor of MACE (adjusted HR 2.30, 95% CI 1.25–4.23; *p* = 0.008), even after adjusting for comorbidities. These findings underscore the prognostic value of arterial calcification for long-term cardiovascular morbidity and mortality in this population. In the study published by Huang et al. (8), patients in the highest calcium score quartile had a 5.16-fold (95% CI 1.13–21.61, *p* = 0.04) increased risk of death compared to those in the lowest quartile. Similarly, annular tibial artery calcification has been identified as a robust predictor of all-cause mortality (HR = 3.19) (28), and complete annular calcification in crural or femoropopliteal arteries independently predicted 10 years mortality (29). Kang et al. (10) also reported that survival free of MACE was significantly higher in the lowest calcification score group (*p* = 0.028). More recently, Megale et al. (15) reported among 72 patients and 88 lower limb revascularizations that the calcium score of the operated limb was higher in patients who died within 30 days and 6 months (6571 vs. 2590.6; *p* = 0.026) and (5227.8 vs. 2335.3; *p* = 0.036). Additionally, in a cohort of 84 patients, higher ICS had significantly lower left ventricular ejection fraction, with a moderate inverse correlation between the two (r = −0.54; *p* < 0.0001) (13). Collectively, these data reinforce the prognostic value of arterial calcification as a marker of systemic cardiovascular risk in this patient population.

As above described, several studies have demonstrated that peripheral arterial calcium scores, including those of the iliac and femoral arteries, are associated with increased severity of PAD and worse cardiovascular outcomes in symptomatic patients (30) and additionally in this study ICS demonstrated to be an independent prognostic value by stratifying long-term cardiovascular and limb-related risk in patients undergoing revascularization for complex aortoiliac disease, suggesting it may serve as a global marker of systemic atherosclerosis. may serve as a reflection of the overall systemic arterial calcification burden. However, the use of these calcium scores as a stratification and prevention tool in asymptomatic and claudicant populations remains underexplored. Future prospective research could focus on the potential value of iliac calcium scoring for early risk stratification and cardiovascular prevention in these patient groups, similar to the role already established for coronary artery calcium scoring in primary prevention settings (31, 32).

This study has several important limitations. Although it was conducted across two centers — a large academic institution and a community referral hospital, which enhances external validity — the sample size remains relatively small. Additionally, the marked predominance of male patients (95.4%) significantly limits the generalizability of findings to the broader population, particularly to women. To mitigate potential selection bias arising from the inclusion of only patients with available CT imaging, a sensitivity analysis was performed (Supplementary Table 1). Second, the *post hoc* design introduces inherent limitations, including the possibility of selection bias: patients with significant comorbidities or unfavorable clinical status may have been more likely to undergo less invasive procedures or to be excluded from revascularization altogether. This is particularly relevant given that current guidelines recommend open surgery for healthier patients with extensive aortoiliac disease. At the same time, an endovascular-first approach is often favored in those with higher operative risk (33). Third, although significant associations were found between calcium scores and clinical outcomes, causality cannot be established due to the study’s observational nature. In addition, variability in the measurement of calcium scores may have influenced patient classification into high-score vs. low-score groups, and absolute score values are known to be affected by age, sex, and ethnicity—factors not fully controlled for in this cohort, despite the limited ethnic diversity in the region. Finally, the pattern of arterial calcification was not evaluated (intima layer vs. media layer) or specific morphological features such as circumferential characteristics or thickness of the artery, which recent studies have identified as potentially relevant predictors of outcomes (28, 29).

Despite these limitations, the study’s strengths include its prospective data collection, extended follow-up period, and the relative homogeneity of aortoiliac lesions, all of which support the robustness of the observed associations. Further prospective cohort studies with larger and more diverse populations are needed to validate these findings.

## Conclusion

This study introduces the ICS as a lesion-specific tool capable of stratifying long-term cardiovascular and limb-related risk in patients undergoing revascularization for complex aortoiliac disease. The ICS demonstrated independent prognostic value beyond perioperative outcomes, highlighting its relevance for long-term clinical decision-making. By quantifying arterial calcification in a standardized manner, the score enhances preoperative risk assessment, informs procedural planning, and supports individualized surveillance strategies. Further prospective cohort studies are needed to confirm the reproducibility and generalizability of these findings across broader populations and to explore the impact of ICS-guided interventions on clinical outcomes.

## Data Availability

The original contributions presented in this study are included in this article/Supplementary material, further inquiries can be directed to the corresponding author.

## Footnotes

1
https://sample-size.net/sample-size-survival-analysis/
