# Supplementary material for: Iliac Calcium Score thresholds predict cardiovascular and limb-related outcomes in TASC D aortoiliac disease
Source: Front Med (Lausanne). 2025 Sep 17;12:1655229. doi: 10.3389/fmed.2025.1655229 (PMC12484223; doi:10.3389/fmed.2025.1655229)
Supplement: Supplementary file 1 [file Table_1.docx]

**Table 1** - Patient’s demographics and comorbidities

| Characteristics | Patients Included  n = 109 (%) | Patients Excluded  n=71 | P value |
| --- | --- | --- | --- |
| Age, years (mean±SD) | 62.0±8.70 | 63.3±9.37 | 0.727 |
| Gender, male | 104 (95.4) | 63 (88.7) | 0.091 |
| Smoking history | 104 (95.4) | 61 (85.9) | **0.024** |
| Hypertension | 76 (69.7) | 52 (73.2) | 0.611 |
| Dyslipidemia | 79 (72.5) | 50 (70.4) | 0.765 |
| Diabetes | 29 (26.6) | 23 (32.3) | 0.402 |
| CKD | 10 (9.2) | 8 (11.3) | 0.647 |
| CAD | 29 (26.6) | 19 (26.8) | 0.982 |
| CVD | 16 (14.7) | 6 (8.5) | 0.212 |
| COPD | 12 (11.0) | 7 (9.9) | 0.806 |
| CHF | 10 (9.2) | 10 (14.1) | 0.306 |

Variables are presented as N (%) unless otherwise specified.

CAD - Coronary artery disease; CHF - Chronic heart failure; CKD - Chronic kidney disease (creatinine ≥ 1.5 mg/dl); COPD - Chronic obstructive pulmonary disease; CVD – Cerebrovascular disease; SD – Standard Deviation. Bold value represents statistically significant results (p < 0:05).
